# Towards an appropriate ethics framework for Health and Demographic Surveillance Systems (HDSS): learning from issues faced in diverse HDSS in sub-Saharan Africa

**DOI:** 10.1136/bmjgh-2020-004008

**Published:** 2021-01-06

**Authors:** Alex Nginyo Hinga, Sassy Molyneux, Vicki Marsh

**Affiliations:** Health Systems and Research Ethics, KEMRI-Wellcome Trust Research Programme, Kilifi, Kenya

**Keywords:** health systems, public health, qualitative study, epidemiology

## Abstract

**Introduction:**

Health and Demographic Surveillance Systems (HDSS) collect data on births, deaths and migration from relatively small, geographically defined populations primarily in Africa and Asia. HDSS occupy a grey area between research, healthcare and public health practice and it is unclear how ethics guidance that rely on a research-practice distinction apply to HDSS. This topic has received little attention in the literature. In this paper, based on empirical research across sub-Saharan Africa, we map out key ethical issues for HDSS and assess the relevance of current ethics guidance in relation to these findings.

**Methods:**

We conducted a qualitative study across seven HDSS sites in sub-Saharan Africa, including individual in-depth interviews and informal discussions with 68 research staff, document reviews and non-participant observations of surveillance activities. Qualitative data analysis drew on a framework approach led by a priori and emergent themes, drawing on the wider ethics and social science literature.

**Results:**

There were diverse views on core ethical issues in HDSS, including regarding the strengths and challenges of community engagement, informed consent and data sharing processes. A key emerging issue was unfairness in the overall balance of benefits and burdens for residents and front-line staff when compared with other stakeholders, particularly given the socioeconomic contexts in which HDSS are generally conducted.

**Conclusion:**

We argue that HDSS operate as non-traditional epidemiologic research projects but are often governed using ethics guidance developed for traditional forms of health research. There is a need for specific ethics guidance for HDSS which prioritises considerations around fairness, cost-effectiveness, ancillary care responsibilities, longitudinality and obligations of the global community to HDSS residents.

**Key questions**

**What is already known?**

- Health and Demographic Surveillance Systems (HDSS) generate data on deaths, births and other health-related events in low-income and middle-income countries where most civil registration and vital statistics systems are incomplete.
- HDSS occupy a grey area between research and practice, which makes it difficult to determine the relevance of traditional ethics guidance that often rely on a research-practice distinction.
- There is limited empirical evidence on ethical issues in HDSS, but the potential social value of longitudinal data from HDSS is thought to outweigh potential burdens.

**What are the new findings?**

- HDSS across sub-Saharan Africa seem to operate as non-traditional epidemiological research projects drawing on widely divergent ethics policies and practices, including for ethics oversight, consenting, community engagement and data sharing.
- HDSS ethics practices and application of traditional ethics guidance, which tend to focus on informed consent and data sharing for research, seem to restrict benefits for HDSS residents.
- From the perspective of HDSS residents, HDSS present important uncompensated burdens.

**What do the new findings imply?**

- In addition to advancing knowledge through research and contributing to global health estimates, HDSS research stakeholders have an ethical responsibility for promoting the greater use of HDSS data to directly benefit HDSS residents and local health information systems.
- There is a need for further empirical research to explore the lived experiences of HDSS stakeholders and to support the development of HDSS-specific ethics guidelines that address ethical issues arising over time at individual, institutional and HDSS population level.

## Introduction

A well-functioning health information system is a valuable resource. It draws from civil registration and vital statistics systems (CRVS), individual medical records, population censuses and other routine sources of individual, institutional and population-level data in a country.^1^ These data are crucial for improving health, protecting human rights, informing social policy and supporting programme evaluation.^2–4^ However, the routine sources of data for health information systems in most low-income and middle-income countries (LMICs) are underdeveloped.^5 6^ Illustratively, a recent analysis of national surveys showed that over half the births (53.3%) of children aged under 5 years in Eastern and Southern Africa were unregistered and less than a third (26.9%) of these children had a birth certificate.^7^

**Figure 1:**
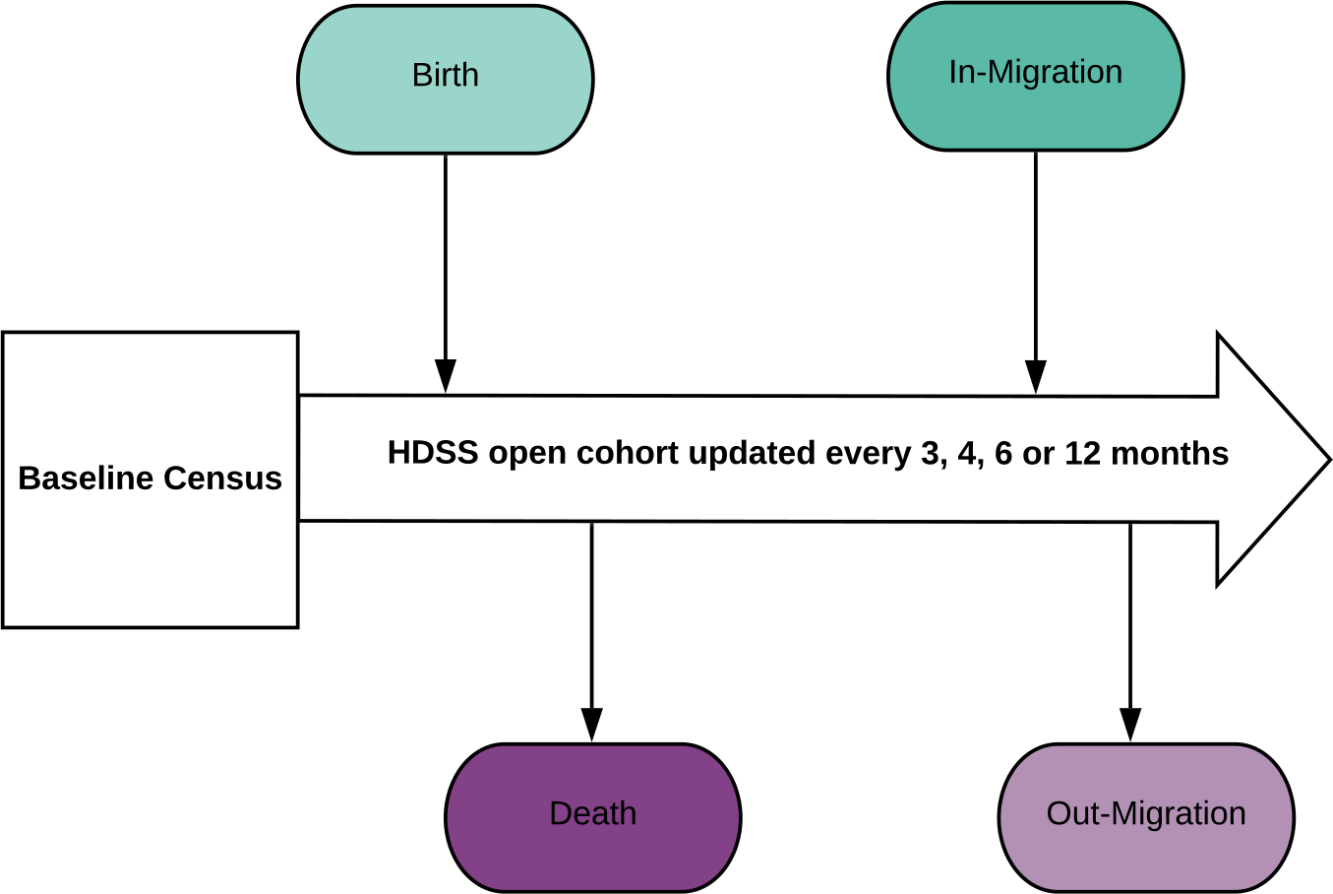
Basic structure and core functioning of a Health and Demographic Surveillance System (HDSS).

Health and Demographic Surveillance Systems (HDSS) are interim sources of health-related data in Africa, Asia and Oceania, where many countries lack well-functioning health information systems.^8 9^ At a minimum, HDSS involve the active and long-term surveillance of births, deaths, cause of death, pregnancies and migration in relatively small geographically defined populations^9 10^ (figure 1). HDSS seek to recruit all residents in the target geographic area and collect data at the individual and household level, mainly during visits to households by field workers, and without a specified end date or official certification of vital events.^8–12^ The frequency of data collection varies across sites, ranging from quarterly^13–15^ to annually.^16 17^ Besides this core functioning, HDSS are often used to collect a wide range of additional public health, clinical and socioeconomic data, including household income, marital status and blood samples, depending on the objectives and interests of each site.^9 10^

Methodologically, HDSS seem to occupy a grey area between research and practice (healthcare and public health).^18^ Scholars have distinguished health research from healthcare or medical practice mainly based on intent; an explicit intention to develop or contribute to generalisable knowledge, including through experimentation, is viewed as a key feature of research while healthcare refers to activities aimed at enhancing the well-being of individual patients using evidence-based or routine methods that are reasonably expected to succeed.^19 20^ Public health practice focuses on the well-being of populations rather than individual patients and includes activities such as surveillance, evaluation and monitoring.^21 22^ An alternative approach to distinguishing research from practice is to consider who runs an activity.^21 23^ For example, some have argued that public health surveillance conducted by public health authorities is not research, even if it involves systematic methods and produces generalisable knowledge.^24^

Historically, health research institutions established HDSS to address specific research questions and to generate longitudinal data for multiple uses, including evaluation^9 25^; online supplemental file 1 details original rationale for establishment of 37 HDSS sites across sub-Saharan Africa. Over time, HDSS have become complex programmes, involving multiple stakeholders and supporting diverse health-related activities.^26^ Some HDSS support healthcare by linking household surveillance data with individual medical records.^15 27 28^ Most HDSS serve as sampling frames and platforms for interventions and nested studies, including community-based health projects,^29^ observational studies and clinical trials.^30^ Overall, the core HDSS functioning and diversity in associated activities contributes to uncertainty on what HDSS are, in methodological terms.

10.1136/bmjgh-2020-004008.supp1 Supplementary data

Traditional research ethics guidance for health-related activities rely on a distinction between research and practice.^19 31^ Widely used examples of health research ethics guidance include the International Ethical Guidelines for Health-related Research Involving Humans^32^ and the Emanuel *et al*^33^ framework for research in developing countries. Widely referenced guidance, focused on public health practice, include the Kass *et al* framework^34^ and reports from the Nuffield Council on Bioethics.^35^ Recently, ethics guidelines and frameworks have been developed for health-related activities that clearly cross the traditional boundary between research and practice, including learning healthcare systems,^36 37^ public health surveillance^38^ and health systems research.^39^ However, it is unclear where HDSS lie on the spectrum of health-related activities and how existing ethics guidance apply.

HDSS have attracted little empirical ethics research despite involving millions of people in LMICs for decades.^18 40^ Most studies have focused on single sites, specific issues or stakeholders, such as perspectives of researchers on research using HDSS data^41^ and specific components of HDSS, including verbal autopsy.^42 43^ Previous empirical ethics studies suggest that community members develop a nuanced understanding of HDSS (eg, greater familiarity and acceptability of core HDSS functions than nested studies) and increased expectations of local and direct benefits after long-term engagement.^44 45^ Some have highlighted that reporting HDSS findings to residents through ‘one-off’ or recurring face-to-face meetings is feasible but requires significant investment of resources.^46 47^ This literature highlights a need for a holistic investigation of ethical issues in HDSS.

In this paper, we describe research that aimed to develop a grounded overview of core ethical issues for HDSS sites in sub-Saharan Africa and to assess the suitability of using different ethics frameworks and guidelines to identify and respond to these issues. This study was informed by the wider ethics literature and perceptions and experiences of stakeholders across the International Network for the Demographic Evaluation of Populations and their Health (INDEPTH Network), formed in 1998 as an organisation of HDSS sites across Africa, Asia and Oceania.^9 10 12^

## Methods

### Study design

We conducted an empirical ethics study combining qualitative social science data collection and analysis drawing on ethics literature to develop normative conclusions^48 49^ on ethical issues for HDSS in sub-Saharan Africa.

### Study setting

Seven diverse INDEPTH Network HDSS sites across five sub-Saharan African countries were included in this study (table 1). Most (41/56) INDEPTH Network HDSS sites are in sub-Saharan Africa where they collectively follow-up over 3.5 million people in 14 countries.^50^ For this reason, and pragmatic considerations such as social access and geographic proximity, we focused on sites in sub-Saharan Africa. We conducted more in-depth work in two Kenyan sites as part of a focused study on verbal autopsy in HDSS, including focus group discussions with HDSS residents, which will be published elsewhere.

**Table 1:** Features of HDSS study sites

Host country	Site urbanicity	Size of site (km^2^)	Population size (approx.)	HDSS inception year
Ghana^98^	Rural	7162	140 000	2003
Ghana^99^	Rural	1675	156 735	1992
Kenya^15^	Rural	891	280 000	2000
Kenya^29 100^	Urban	5 - 6.5	88 974	2002
Malawi^67^	Rural	135	39 000	2002
South Africa^28^	Rural	438	139 250	2000
Uganda^27^	Rural	28	23 000	1989

HDSS, Health and Demographic Surveillance Systems.

### Data collection

Data were collected through interviews and informal discussions with INDEPTH Network secretariat members, health workers in facilities affiliated with HDSS sites, and with HDSS researchers, managers and field workers, with the latter referring to HDSS field supervisors, routine census and verbal autopsy interviewers (table 2). We complemented these interviews and discussions with document reviews and non-participant observations involving HDSS field workers, residents and community representatives.

**Table 2:** Characteristics of interview and informal discussion participants

	N		
	Individual interviews	Informal discussions	Total
Gender			
Male	17	21	38
Female	12	18	30
Roles in HDSS			
Researcher	12	13	25
Manager	10	18	28
Field worker	7	5	12
Health worker	0	3	3
Affiliations			
Kilifi	15	1	16
Nairobi	9	6	15
Karonga	1	6	7
Kintampo	1	5	6
Kyamulibwa	2	10	12
Navrongo	0	7	7
AHRI	1	1	2
INDEPTH Network	0	3	3
Overall total			68

HDSS, Health and Demographic Surveillance Systems.

#### Individual interviews with HDSS research staff

Individual in-depth interviews (IDIs) were conducted through face-to-face meetings in Kenya and telephone or online video calls for participants in other settings. The interview guides included open questions about the participants’ responsibilities in the HDSS, views and experiences around HDSS methodological design, ethics review, consenting, community engagement, data and benefit sharing, and other ethically relevant issues. All the interviews were digitally recorded. The average duration of each interview was 58 min.

#### Informal discussions and overt non-participant observations of surveillance activities

Informal discussions^51^ were held with individual HDSS research staff and administrators purposively selected based on their ability to share views and experiences of working in HDSS. These discussions were held in natural settings, including participants’ homes and offices.

Non-participant observations^52^ were undertaken during HDSS census and verbal autopsy interviews in 59 households, a field worker training workshop on electronic data collection and four meetings between HDSS research staff and community representatives. The observer (ANH) played no role in HDSS data collection or workshop facilitation and participants were made aware of the observations and the HDSS ethics study. Given the informality of these discussions and observations, there were no audio recordings, but notes were taken throughout.

#### Document reviews

Information about the characteristics of HDSS sites in sub-Saharan Africa was obtained from the INDEPTH Network website. Unpublished documents from the selected HDSS sites, including information and consent forms, HDSS questionnaires, workshop reports, and data and benefit sharing guidelines, were reviewed. Documents and quantitative data accessed in the Kenya sites for an in-depth verbal autopsy study, described elsewhere, provided additional insights into HDSS community engagement and data sharing processes.

### Data management and analysis

Qualitative data were managed using the NVivo V.10 and analysed using the framework approach.^53^ This process involved transcription of audio recordings, an in-depth familiarisation with content and a team approach to independently developing coding frameworks before identifying an agreed schema around HDSS characteristics, ethical policies, practices and perspectives across different sites and participant groups. All authors interpreted these data, drawing from the wider ethics and social science literature, to identify the key characteristics and core ethical issues for HDSS in sub-Saharan Africa.

### Ethical considerations

Verbal consent was obtained for informal discussions, non-participant observations and interviews held through telephone and video calls. Written consent was obtained for face-to-face interviews. The study was reviewed and approved by the relevant ethics committees (box 1). In the Findings section, individual codes are not linked to sites where roles would risk identification.

Box 1 **Scientific and ethics committees involved in this study**

1. KEMRI Wellcome Trust Research Programme Centre Scientific Committee
2. KEMRI Scientific and Ethics Review Unit (SERU)
3. Uganda Virus Research Unit Research Ethics Committee
4. Uganda National Commission for Science and Technology
5. London School of Hygiene and Tropical Medicine Ethics Committee
6. Malawi National Health Sciences Research Committee
7. Kintampo Health Research Centre Scientific Review Committee
8. Kintampo Health Research Centre Institutional Ethics Committee
9. Navrongo Health Research Centre Institutional Review Board

## Results

We describe empirical findings related to HDSS methodological design, processes of ethics review, community engagement, informed consent, data sharing, and benefits and burdens in turn. While data on these themes were gathered from all sites, there is more in-depth data from sites 1 and 2. Where differences in views on ethical issues or, more commonly, practices were observed across sites, these are highlighted.

### Views on HDSS methodological design

To contextualise views on ethical issues and given the indistinct positioning of HDSS, we sought to find out how participants defined HDSS beyond describing associated activities. Research staff, including those working in the same sites, often had diverse views on the appropriate methodological definition of HDSS, with some defining HDSS as ‘research’ or ‘platforms for research’ and others defining them as ‘not research’.

I have the view that since it (HDSS) was set up for research purposes, it should be treated like any other research activity or project. Researcher_Site 4

I would bill it as surveillance rather than research. It doesn’t have a research question itself. Research questions are bolted onto it, it is a sort of platform onto which research projects happen. HDSS-Manager_Site 1

A third and the largest group of participants felt that the definition of HDSS was unclear or highly context specific.

I think the main classification is dependent on who constitutes the DSS. DSSes that are constituted by governments, in my view, are involved in public health. I think a majority are constituted by research institutions, those I think are more or less research. Researcher_Site 1

Based on field observations, including conversations between HDSS residents and field workers, most residents seemed to see HDSS activities as conducted by a research institution and therefore largely as a research activity. However, some residents, especially in sites affiliated with healthcare facilities, also described these institutions as health service providers.

When defining HDSS, as illustrated above, most research staff discussed (even before further prompting) the implications of this definition for ethics review and consenting processes, discussed next.

### HDSS ethics review processes

Ethics oversight processes in the HDSS sites studied ranged from one time approvals from government (without formal ethics review), through just one initial formal review by a research ethics committee (REC), to annual REC reviews.

From the outset, we have always applied for ethics approval from the (National ethics review committee). Every time we have a new tract of funding, when we introduce new procedures, we apply for approval. We also send annual updates to the ethics committee for renewal. HDSS-Manager_Site 3

…for other protocols even if they get ethics approval it is only valid for a year and then there are continued protocol review and ethics renewal. With the DSS, that is not what happens, after we renewed in 2011, that was that. Researcher_Site 4

However, changes in international ethics policies and guidelines around health-related research were reportedly influencing HDSS to submit protocols to research ethics review committees for annual ethics review and approval.

Many who supported research ethics review for HDSS cited a regulatory rationale, pointing out, for example, that ethics approval would facilitate publication of HDSS findings in scientific journals. Others cited more fundamental ethical protections, such as the role of ethics review in protecting residents from potentially harmful procedures.

### HDSS community engagement

Across the seven sites, decisions to establish HDSS were preceded by information sharing, consultations and partnership building with a wide range of stakeholders, including government and community members.

…there was a lot of communication with the stakeholders… the community, chiefs, the sub-chiefs, the civil registration departments, the national bureau of statistics, they were all positive about this kind of work. There was a lot of internal consultations and discussions to okay the DSS to start. HDSS-Manager_Site 1

As an ongoing activity, HDSS community engagement is largely concerned with providing information to residents through mass media platforms, community meetings and distribution of pamphlets in the HDSS areas. Other forms of HDSS community engagement have been included, such as community consultation and—in three sites in this study—key informant systems, in which community members collect some HDSS data.

Notably, most of these community engagement activities, such as radio programmes or community meetings, are not HDSS-specific; instead, they are embedded in large one-off community engagement initiatives supporting a range of HDSS-linked research activities led by the host institutions. Relatedly, some field workers felt that community engagement and other HDSS-specific issues had not received adequate attention.

…sincerely speaking, since (studies nested within HDSS platforms) came on board, I think the centre has a lot of priorities. So, we don’t really get attention. It’s been more than ten years since we did dissemination for our DSS. VA-Interviewer_Site 2

### Seeking consent from HDSS residents

In HDSS, consent for routine data collection is obtained at the household level.^18^ Experiences from site 1 highlighted the practical challenges of obtaining individual informed consent for routine HDSS census. In this site, a stakeholder described a pilot study that was conducted to assess feasibility of obtaining individual verbal consent from all adults (>=18 years) and mature minors, and verbal assent from children aged 13 to 17 years.

…that approach never worked. It turned out to be expensive because you need to have almost five times the fieldworkers here. HDSS-Manager_Site 1

Field workers in four out of the seven sites obtained verbal consent from HDSS residents, while written consent was the approved form of documenting consent in three sites. Support for verbal consent was common across many field workers and managers. This position was argued on the basis that participating in a routine HDSS census interview is a low-risk activity, and that written consent could lead to unnecessary refusals, raise tensions, present logistical challenges and undermine trust, especially in contexts where residents are likely to associate signing forms with legal and financial transactions. Those who supported written consent argued this on a procedural basis, that is, that it could provide documentary evidence that field workers had collected data with consent from residents. While many participants felt verbal consent would be most appropriate, they also felt this would not generally be acceptable to some ethics review committees.

(verbal consenting) is not acceptable to the regulatory authorities. You have to make a very, very strong case for verbal consenting, it is very rare, I can get it (approval for verbal consenting) for some of the social science observations but not with a questionnaire. HDSS-Manager_Site 5

Ethics review committees and national laws may also prescribe written consent for HDSS because HDSS collect a wide range of data under the same protocol.

We have a number of components in the HDSS… The (law) requires we obtain consent for collecting certain data such as telephone contacts, for us to prove legally that we obtained consent we need it to be written. So, to make our work smooth we decided that we needed written consent for every aspect of our data collection. HDSS-Manager_Site 7

Field observations highlighted additional barriers to informed consenting in HDSS, including uncertainty on which information is necessary, feasible and culturally appropriate to share with HDSS residents at the time of data collection, given that HDSS involve multiple procedures and stakeholders. Acknowledging these challenges, some participants recommended that the consenting process would be better seen as part of a wider effort to build mutual understanding and show respect between HDSS stakeholders, than as a stand-alone activity.

The consent itself should begin with the homestead head if possible then go to each of the households within the homestead…explain to individuals within households…and back all of that up with a really good communication and community engagement strategy. Researcher_Site 1

### HDSS data sharing and use

The INDEPTH Network has platforms for HDSS data sharing at the international level,^54^ but data sharing policies and practices vary across sites. While some sites provide email addresses as a contact to request data, others have online data repositories and detailed institutional data sharing policies that outline the ethical reasons for sharing and procedures for requesting data.

Our data is put in a public repository. There are rules regulating the repository, but basically freely available to whoever wants to rationally use the data. Whether [it’s] individuals, institutions or agencies they will be able to access the data. HDSS-Manager_Site 7

Discussions with participants and field observations highlighted practical barriers to HDSS data sharing, including limited human resources for effective and timely data entry and cleaning. Also, some felt that data sharing could have unintended consequences, such as reducing data quality by disincentivising data collection, and promoting unfairness among stakeholders. Unfairness was seen both in terms of the interests of primary researchers and those who later use data, and between primary researchers and local and national HDSS stakeholders. In the latter case, researchers and HDSS managers reported using HDSS data for research, but all participants acknowledged that other stakeholders, including policy makers, field workers and community members might find it difficult to access and use HDSS data.

… I remember there was someone who wanted to develop a proposal to apply for government funds for youth… I am just imagining that if they wanted even simple statistics such as population size, how would they access it? Because I don’t think they even have internet or anything, they write the proposals by hand. Researcher_Site 2

### Benefits: generating data for research, policy and public health

The most prominent benefit of HDSS, from the perspective of HDSS stakeholders, was an aspiration that HDSS data would contribute to improvement of public health by supporting the responsiveness of future research and policy making. As an illustration, one HDSS information and consent form states that HDSS data “… *will help the government plan public health services…*”. Participants also described some potential benefits for the local community, including direct and indirect employment.

Discussions with participants, and field observations, suggested that HDSS interviews could be positive experiences for some HDSS residents and field workers, in supporting friendly and interesting interactions. Further benefits reported were the renovation and establishment of local health facilities, healthcare and occasional provision of various forms of appreciation, such as soap or water purification tablets.

When a participant reports to the clinic with some ailments, and we find we cannot help them here… we refer and transport them to the referral hospital, and if necessary, we meet some of the costs for their treatment. HDSS-Manager_Site 5

### Burdens of HDSS sites in sub-Saharan Africa

Potential burdens were identified at different levels and for different stakeholders. The most prominent burdens are related to HDSS residents and field workers. Time costs and ‘fatigue’ for HDSS residents emerged as the most reported burdens, where ‘fatigue’ was used to describe a sense of tiredness, boredom and some resentment towards repeated visits, against a background of few direct benefits for families involved. From observation, the duration of HDSS interviews varied, being particularly long in large households and where field workers used paper-based questionnaires or collected additional data, such as vaccination history. Also, most HDSS residents needed to abandon various activities (such as farming, washing clothes and vending food) to take part in the interviews.

…some of them feel that we have collected these data so much that they are now tired. VA-interviewer_Site 2

In addition, some participants pointed to risks to HDSS residents’ privacy and confidentiality through data collection and management, with risks seen as occurring at a very local level (within the institution) as well as the risks of data sharing more widely, as highlighted earlier.

…Although the DSS itself does not contain any particular revelations…there are obviously contentious issues of knowing paternity, people living in houses which perhaps they shouldn’t be, perhaps they are not married… and then within the (research institution) we have people who are able to see data, names and places on databases and potentially that could be misused if it got in the wrong hands. HDSS-Manager_Site 1

Similarly, field observations suggested that questions around pregnancy and marital status, death, source and amount of household income, and ownership of economic assets, pose risks to privacy and could make some residents uncomfortable. However, the most severe burden described across all sites, as will be described in detail in a future publication, was emotional distress for HDSS residents and field workers directly involved in verbal autopsy. The verbal autopsy entails interviewing close relatives or final carers of the deceased to establish the circumstances and likely cause of death.^55 56^ The verbal autopsy is a much less frequent occurrence at the household level and methodologies may vary across sites, but inclusion of this activity is a current requirement for HDSS to be part of INDEPTH, given the potential public health value of cause of death data.^2 26^

If I was to grade sensitivity of HDSS data, I would give socioeconomic data 6 out of 10 and Verbal Autopsy 10/10. HDSS-Manager_Site 2

Burdens associated with HDSS that were less commonly mentioned included insecurity and economic costs for HDSS field workers, who may feel compelled to make out-of-pocket financial contributions to assist residents facing significant health and socioeconomic challenges, for example. Other burdens included risks of supplanting local health systems, a potential for stigmatisation of HDSS communities (particularly in relation to the reporting of patterns of stigmatising illness or socioeconomic status across identifiable communities).

There was a time when the DSS was collecting data on toilet ownership, the community members came here (research institution) and we presented that data, there was a location that didn’t have a toilet at all…everyone turned their heads (to look at people from that location) … after that the chief came to complain…‘you’ve made us feel like idiots’. Researcher_Site 1

In table 3, we draw from prominent research and public health ethics frameworks^33 34 57 58^ to summarise the core ethical issues emerging from our findings, alongside their ethical implications.

**Table 3:** Key ethical issues in Health and Demographic Surveillance Systems

Theme	Ethical considerations	Ethical issues
Establishing a HDSS	Have the HDSS area and populations been selected fairly? The need for collaborative partnerships.	Risks of selecting the most vulnerable areas and populations. Most HDSS are in rural and poor urban areas of sub-Saharan Africa and have limited integration with CRVS and other health information systems.
HDSS methodological design	What are the objectives of the HDSS and how effective is the HDSS in meeting these objectives?	Unclear objectives, including that initial objectives of most HDSS have not been updated despite changes in HDSS functioning over time. Risks of collecting incomplete or inaccurate data for example, where insufficient funding and cultural sensitivities may hinder regular enumerations and collection of accurate data on income, pregnancy status and cause of death.
Ethics review processes	What ethical principles, theories and guidelines apply to the HDSS?	There is uncertainty over the appropriate ethics guidance for HDSS, leading to: Ethics review processes being inconsistent across sites, including no annual ethics review and approval. Default use of biomedical research ethics guidelines focusing on time limited individual-level issues only, when HDSS follow populations over time. Practices of self-regulation, exempting harmful procedures from independent review. Ethics review processes being unnecessarily burdensome.
Community engagement	Is community engagement necessary? What community engagement activities are feasible/appropriate?	Use of poorly defined concepts, such as ‘HDSS community’ and insufficient resources for HDSS-specific community engagement, leading to risks that community engagement is unduly limited for example, information sharing only.
Informed consent	Respect for individual autonomy and local community	Tensions between individual autonomy and enhancing social value, where individual written informed consent processes likely to compromise HDSS data quality and increase HDSS operating costs and burdens for residents and field workers (eg, interpretations of why a signature is needed may lead to refusals). Procedures and feasibility of withdrawing from HDSS are unclear, which may limit this choice.
Data sharing and reporting Results	What are the appropriate HDSS data governance systems? Who should access and use HDSS data?	HDSS collect, link, analyse and disseminate a wide range of sensitive data, generating potential risks to privacy and confidentiality including community stigmatisation from reporting sensitive community-level findings Risk of damaging trust among HDSS stakeholders Limited use of HDSS data
Benefits and burdens	What are the benefits of HDSS and who are the beneficiaries? What are the burdens of HDSS? Can they be minimised? Are burdens justified?	Risks of defining benefits narrowly, so that non-health benefits and beneficiaries of HDSS are unspecified Inability to measure and enhance benefits or identify, weigh and respond to HDSS burdens. Female residents, verbal autopsy respondents and interviewers often bear most burdens (time, insecurity, emotional distress) and little to no benefits. Since HDSS data are mainly used for research purposes, researchers and global health modellers who gain direct benefits from using HDSS data (influence, career development, funding) bear the least burdens. Overall risks of a disproportionate distribution of burdens.

CRVS, civil registration and vital statistics systems; HDSS, Health and Demographic Surveillance Systems.

## Discussion

The empirical findings from this study identify important ethical issues associated with the conduct of HDSS (table 3) and highlight a core emerging ethical issue of fairness in the benefits and burdens experienced by HDSS stakeholders. These issues are not unique to HDSS and are partially addressed by different ethics frameworks and guidelines for research, public health and other health-related activities. However, the use of these frameworks and guidelines to address ethical issues in HDSS presents significant conceptual and practical challenges because in practice HDSS operate as what we describe as ‘non-traditional research’. There is a recognised need for better ethics guidance in non-traditional areas of public health and epidemiology.^59^ The development and application of specific ethics guidance for HDSS could highlight the main ethical issues and trade-offs and outline core ethical principles. We draw on our empirical findings, current ethics frameworks and guidelines, and the wider ethics literature to discuss ethical issues in HDSS and to contribute towards the development of an appropriate ethics framework for HDSS.

### Community engagement in HDSS

Across our findings, and in common with others, we have highlighted conceptual and practical challenges for community engagement in HDSS.^46 47 60 61^ Reliable funding for HDSS can address some practical challenges, but addressing the conceptual challenges is more complex. The first conceptual challenge is that while a HDSS community is a geographically defined ‘community’, it does not include all residents as some can refuse to take part and others may not meet site-specific inclusion and exclusion criteria. There are also varying levels of participation in HDSS, for example, women are likely to be the main respondents for HDSS interviews. Therefore, a geographic-based definition of HDSS community seems inadequate. The concept of ‘experimental publics’ was developed to challenge the perception that a study community is a group of people with shared characteristics that pre-exist research,^62^ as might be argued for a HDSS. Instead, experimental publics are created through study procedures, such as the application of inclusion and exclusion criteria.^44 62^ A crucial issue in HDSS is that HDSS operate without a specified end date, with important implications for communities, relationships and costs, but current ethics guidance addresses this issue only superficially.

A second core conceptual challenge for HDSS community engagement concerns the meaning of ‘engagement’, with different goals of engagement reflecting different levels of power-sharing between HDSS practitioners and community members. While there was variation between sites, most of the engagement activities reported in this study involved information sharing as opposed to seeking community input into HDSS design or policy. In addition to the existing ethics guidance on establishing collaborative partnerships with communities and their leaders,^33 61^ an ethics framework that views HDSS communities as experimental publics could support the development of more tailored engagement strategies.

### Informed consent processes in HDSS

Our findings add to an extensive literature that highlights ways in which informed consent processes in research in sub-Saharan Africa are significantly impacted by wider health and social factors, including interpersonal relationships, cultural norms, expectations of health benefits and low literacy.^45 63–65^ In the sites involved in this study, field workers generally sought informed consent at the household level because obtaining consent from each resident for routine HDSS activities was impractical. Many study participants supported verbal consenting. A challenge is that some research ethics committees, drawing from traditional ethics frameworks, may not waive requirements for written consent unless they view HDSS as research with high social value and minimal risks,^32^ or as public health practice^66^; conditions that many HDSS might not meet.

The high consent rates reported in HDSS^67–70^ seem to indicate general community acceptability, but they are likely to be a crude measure of community understanding, voluntariness or burdens experienced by HDSS residents, given general concerns with informed consenting in these contexts. In HDSS within sub-Saharan Africa, a requirement for written consent may not achieve intended ethical goals, and potentially increases practical and emotional burdens for HDSS respondents, field workers and institutions. HDSS-specific community engagement could strengthen consenting practices, while effective data governance could achieve the ethical goals of consenting without requiring HDSS residents to sign forms. A non-traditional ethics framework that acknowledges the unique features of HDSS methodology and ethical tensions between individual autonomy and population-level benefits might allow adaptation of consenting to specific HDSS contexts, in contrast to traditional research ethics guidance that is likely to prescribe written consent.

### Data sharing and use

The principal goal in sharing health research data—and for HDSS—is promoting public health interests, including through supporting future research. The ethical and practical barriers to research data sharing in LMICs are well-acknowledged, such as challenges around prior informed consent for uncertain future use, privacy risks for participants, risks of primary researchers being ‘scooped’ by secondary research teams (often reflecting structural inequities in research resources) and concerns about fairness in balancing benefits for those who contribute and those who use data.^71–73^ While most of these challenges apply, HDSS data are freely available online, including through INDEPTH Network, which should promote their social value. At the same time, researchers in sub-Saharan Africa may not have had the capacity building opportunities and resources of other researchers to support analysis of freely available data; a focus on data sharing over data use could exacerbate inequalities.^74^

As other scholars have noted, HDSS data are primarily shared and used for research,^10 75^ with less attention to sharing data with HDSS residents, local policy makers or healthcare providers. This may contribute to a disproportionate distribution of benefits among HDSS stakeholders and inefficient use of resources. For example, most countries have classified CRVS as essential services to continue collecting vital data during the COVID-19 pandemic.^76^ Additionally, international ethics guidelines emphasise that ethical public health surveillance^38^ and research,^77^ including timely data collection and sharing, are crucial for responding to public health emergencies, and yet, some HDSS sites are likely to have stopped collecting data during the COVID-19 pandemic, despite the urgent need for timely data on deaths, migrations and causes of death. There is a strong ethical argument for using HDSS data and platforms to address a wider range of local and international priorities, such as rapid response to public health emergencies. An ethics framework that prioritises fairness could allow greater use of HDSS data, especially for direct benefits to communities that contribute these data.

### Highlighting issues of fairness for benefits and burdens of HDSS among stakeholders

As noted earlier, the justification of HDSS relates to a putative social value that outweighs potential burdens. To some extent, the social value of HDSS is evident, including contributions to global health estimates, provision of longitudinal data^54^ and supporting important public health research in LMICs.^30 78–83^ However, many LMICs lack the infrastructure to convert these research and global health estimates into policy and health gains at the local and national levels.^84 85^ In addition, despite recommendations that HDSS should integrate with other health and population information systems, such as CRVS, to enhance direct and local benefits,^26^ HDSS largely operate independently and for research. Although we identified some potential direct benefits for HDSS individuals and local communities, these were highly context specific. Thus, HDSS provide potentially valuable research evidence and data, but their social value at the local and national levels in sub-Saharan Africa is unclear.

Regarding burdens of HDSS, our study highlights that many HDSS residents and field staff are likely to experience a wide range of often minor burdens over a long time. The verbal autopsy, a core component of HDSS for collecting cause of death data, generates the most severe social and emotional burdens for specific groups of HDSS field staff and bereaved families, and will be the focus of a future publication. At the same time, ethics processes such as ethics review and consenting could unintentionally increase burdens for HDSS stakeholders without protecting HDSS residents.

Overall, the study strongly suggests a high risk that the distribution of benefits and burdens among HDSS stakeholders in sub-Saharan Africa is disproportionate. While stakeholders who can use HDSS data, such as researchers and global health modellers, are likely to gain the most benefits, those who contribute and collect data, such as HDSS residents and field staff, bear the most burdens. We join others in recommending that research ethics review should be adaptive to consider a wider range of ethical issues, principles, stakeholders and study designs.^36 39 86 87^ In relation to HDSS in sub-Saharan Africa, ethics review should prioritise ethical considerations around fairness (taking account of structural inequities evident in HDSS settings), cost-effectiveness, ancillary care responsibilities, obligations of the global community to HDSS residents and HDSS longitudinality.

### Why HDSS are best considered as non-traditional health research

The research-practice distinction has a significant influence on ethics oversight for health-related activities. Activities defined as practice are generally exempt from ethics review while those defined as research are typically subjected to an ethics review process.^19 31^ Although some have observed that HDSS do not fit within the traditional definitions of health research or practice,^18^ little attention has been paid to what HDSS are in methodological terms. This ambiguity risks ethics guidance meant for other activities, particularly biomedical research, being applied to HDSS, which may unduly prioritise individual autonomy and individual-level issues^58 63 88^ over other important ethical considerations in HDSS.

We argue that INDEPTH Network HDSS sites are best considered as ‘non-traditional epidemiologic research’. Epidemiology is the study (including surveillance) of the distribution and determinants of health-related states or events (such as causes of death) to improve health of populations.^89^ An epidemiologic study design can involve the repeated observation of an entire geographically defined population.^90^ While epidemiology overlaps considerably with human subjects research and public health,^59^ it involves practices and values that distinguish it as an academic discipline, including use of specific terminologies, and institutional manifestation.^91^ Terms and methodologies such as ‘cohorts’ and ‘population pyramid’, which are common in epidemiology^89^ are often used in HDSS. Also, most HDSS managers have academic training and expertise in epidemiology. In addition, HDSS cohort profiles are primarily published in journals specialising in epidemiology. Unlike typical epidemiologic research, however, HDSS do not have a specified end date, involve a real-life population rather than subgroups and support a wide range of additional health-related activities.^9 12^ These definitions and standards, coupled with empirical evidence of core functioning, objectives and perspectives in diverse sites in sub-Saharan Africa, strengthen the argument for considering HDSS as non-traditional epidemiologic research.

Some have argued that the research-practice distinction has no independent moral value because defining an activity does not justify why it should be subjected to or exempted from ethics oversight.^19 92^ Instead, some suggest the risks and burdens of a health-related activity should inform ethics oversight processes.^93^ Nevertheless, that the research-practice distinction remains prominent and has value in ethics debate and contemporary ethics practice is exemplified by a recent controversy over whether a WHO Malaria vaccine programme in Ghana, Kenya and Malawi is a cluster randomised trial in breach of international research ethics standards or a public health activity adhering to relevant regulations and widely accepted practice.^94–96^ We suggest that clarifying definitions for HDSS therefore seems to be an ethically important project in its own right.

### Strengths and limitations

We conducted empirical work in seven INDEPTH Network sites, with in-depth data collection in two Kenyan sites. We acknowledge that generalisation of findings from qualitative research can be contested.^97^ However, strength of our data and supporting the transferability of the learning is the diversity of sites, the bringing together of empirical evidence with wider social science and ethics literature, and the shared core methodological approach and standardised procedures across INDEPTH Network sites (figure 1).

## CONCLUSION

Across this paper, we have identified a range of potential ethical issues for HDSS in sub-Saharan Africa and argued that, based on core HDSS design, practices and perspectives across diverse sites, as well as the research methods literature, we should consider HDSS as non-traditional epidemiological research. Adopting this approach and developing an ethics framework specifically for HDSS should address important ethical issues in HDSS, including challenges linked to use of traditional research ethics oversight processes. The most prominent of these ethical issues is the disproportionate distribution of benefits and burdens among HDSS stakeholders. Other key issues include balancing the optimal use of HDSS data, which are collected at significant cost to individuals and institutions, with protecting the interests of stakeholders. Besides mapping out key ethical issues in HDSS (table 3), we have provided empirical evidence of risks and burdens in diverse sites. This contribution is likely to strengthen ethics oversight processes for HDSS.

In addition to developing an ethics framework for HDSS, we need further research in diverse sites and consultation with stakeholders to promote ethical practices and policies. In-depth research around verbal autopsy and HDSS longitudinality, and consultations involving research staff and community members, could further inform HDSS-specific international ethics guidelines.

## References

[1] Greenwell F, Salentine S Health Information System Strengthening: Standards and Best Practices for Data Sources - MEASURE Evaluation. Chapel Hill, NC, 2018.

[2] Sankoh O, Dickson KE, Faniran S, et al Births and deaths must be registered in Africa. Lancet Glob Health 2020;8:e33–4. 10.1016/S2214-109X(19)30442-5 31839137

[3] Setel PW, Macfarlane SB, Szreter S, et al A scandal of invisibility: making everyone count by counting everyone. Lancet 2007;370:1569–77. 10.1016/S0140-6736(07)61307-5 17992727

[4] Brolan CE, Gouda HN, AbouZahr C, et al Beyond health: five global policy metaphors for civil registration and vital statistics. Lancet 2017;389:1084–5. 10.1016/S0140-6736(17)30753-5 28322806

[5] Walsham G Health information systems in developing countries: some reflections on information for action. Inf Technol Dev 2020;26:194–200. 10.1080/02681102.2019.1586632

[6] Mikkelsen L, Phillips DE, AbouZahr C, et al A global assessment of civil registration and vital statistics systems: monitoring data quality and progress. Lancet 2015;386:1395–406. 10.1016/S0140-6736(15)60171-4 25971218

[7] Bhatia A, Ferreira LZ, Barros AJD, et al Who and where are the uncounted children? Inequalities in birth certificate coverage among children under five years in 94 countries using nationally representative household surveys. Int J Equity Health 2017;16:148. 10.1186/s12939-017-0635-6 28821291 PMC5562988

[8] Hill K, Lopez AD, Shibuya K, et al Interim measures for meeting needs for health sector data: births, deaths, and causes of death. Lancet 2007;370:1726–35. 10.1016/S0140-6736(07)61309-9 18029005

[9] Sankoh O, Byass P The indepth network: filling vital gaps in global epidemiology. Int J Epidemiol 2012;41:579–88. 10.1093/ije/dys081 22798690 PMC3396316

[10] Ye Y, Wamukoya M, Ezeh A, et al Health and demographic surveillance systems: a step towards full civil registration and vital statistics system in sub-Sahara Africa? BMC Public Health 2012;12:741. 10.1186/1471-2458-12-741 22950896 PMC3509035

[11] de Savigny D, Renggli S, Cobos D Maximizing Synergies between Health Observatories and CRVS : Guidance for INDEPTH HDSS Sites and CRVS Stakeholders 2018.

[12] INDEPTH Network Population and health in developing countries: population, health, and survival at indepth sites. Ottawa: International Development Research Centre; 2002.

[13] Jasseh M, Gomez P, Greenwood BM, et al Health & Demographic Surveillance System Profile: Farafenni Health and Demographic Surveillance System in The Gambia. Int J Epidemiol 2015;44:837–47. 10.1093/ije/dyv049 25948661

[14] Odhiambo FO, Laserson KF, Sewe M, et al Profile: the KEMRI/CDC Health and Demographic Surveillance System--Western Kenya. Int J Epidemiol 2012;41:977–87. 10.1093/ije/dys108 22933646 PMC12083774

[15] Scott JAG, Bauni E, Moisi JC, et al Profile: the Kilifi health and demographic surveillance system (KHDSS). Int J Epidemiol 2012;41:650–7. 10.1093/ije/dys062 22544844 PMC3396317

[16] Pison G, Douillot L, Kante AM, et al Health & demographic surveillance system profile: Bandafassi Health and Demographic Surveillance System (Bandafassi HDSS), Senegal. Int J Epidemiol 2014;43:739–48. 10.1093/ije/dyu086 24836327

[17] Alberts M, Dikotope SA, Choma SR, et al Health & Demographic Surveillance System Profile: The Dikgale Health and Demographic Surveillance System. Int J Epidemiol 2015;44:1565–71. 10.1093/ije/dyv157 26275454

[18] Carrel M, Rennie S Demographic and health surveillance: longitudinal ethical considerations. Bull World Health Organ 2008;86:612–6. 10.2471/BLT.08.051037 18797619 PMC2649449

[19] Kass NE, Faden RR, Goodman SN, et al The research-treatment distinction: a problematic approach for determining which activities should have ethical oversight. Hastings Cent Rep 2013;Spec No:S4–15. 10.1002/hast.133 23315895

[20] Department of Health, Education, and Welfare, National Commission for the Protection of Human Subjects of Biomedical and Behavioral Research The Belmont report. ethical principles and guidelines for the protection of human subjects of research. J Am Coll Dent 2014;81:4–13. 25951677

[21] Hodge JG An enhanced approach to distinguishing public health practice and human subjects research. J Law Med Ethics 2005;33:125–41. 10.1111/j.1748-720X.2005.tb00215.x 15934670

[22] Griffiths S, Jewell T, Donnelly P Public health in practice: the three domains of public health. Public Health 2005;119:907–13. 10.1016/j.puhe.2005.01.010 15979112 PMC7111730

[23] Remme JHF, Adam T, Becerra-Posada F Surveillance or research: what’s in a name? Sci Adm 2010;3:16.

[24] Lee LM Public health data collection and implementation of the revised common rule. J Law Med Ethics 2019;47:232–7. 10.1177/1073110519857278 31298106

[25] Ferrie JE The irresistible rise of the cohort profile. Int J Epidemiol 2012;41:899–904. 10.1093/ije/dys119 23097761

[26] Sankoh O, Byass P, INDEPTH Network CHESS: an innovative concept for a new generation of population surveillance. Lancet Glob Health 2015;3:e742. 10.1016/S2214-109X(15)00180-1 26511039

[27] Asiki G, Murphy G, Nakiyingi-Miiro J, et al The general population cohort in rural south-western Uganda: a platform for communicable and non-communicable disease studies. Int J Epidemiol 2013;42:129–41. 10.1093/ije/dys234 23364209 PMC3600628

[28] Tanser F, Hosegood V, Bärnighausen T, et al Cohort profile: Africa centre demographic information system (ACDIS) and population-based HIV survey. Int J Epidemiol 2008;37:956–62. 10.1093/ije/dym211 17998242 PMC2557060

[29] Wamukoya M, Kadengye DT, Iddi S, et al The Nairobi urban health and demographic surveillance of slum dwellers, 2002–2019: value, processes, and challenges. Glob Epidemiol 2020;2:100024 10.1016/j.gloepi.2020.100024

[30] Sankoh O, Welaga P, Debpuur C, et al The non-specific effects of vaccines and other childhood interventions: the contribution of indepth health and demographic surveillance systems. Int J Epidemiol 2014;43:645–53. 10.1093/ije/dyu101 24920644 PMC4052142

[31] Beauchamp TL, Saghai Y The historical foundations of the research-practice distinction in bioethics. Theor Med Bioeth 2012;33:45–56. 10.1007/s11017-011-9207-8 22258893

[32] CIOMS and WHO CIOMS guidelines 2016, 2016 Available: http://www.cioms.ch/ethical-guidelines-2016/ [Accessed 13 Jan 2017].

[33] Emanuel EJ, Wendler D, Killen J, et al What makes clinical research in developing countries ethical? The benchmarks of ethical research. J Infect Dis 2004;189:930–7. 10.1086/381709 14976611

[34] Kass NE An ethics framework for public health. Am J Public Health 2001;91:1776–82. 10.2105/AJPH.91.11.1776 11684600 PMC1446875

[35] Nuffield Council on Bioethics Public health: ethical issues; 2007.

[36] McLennan S, Kahrass H, Wieschowski S, et al The spectrum of ethical issues in a learning health care system: a systematic qualitative review. Int J Qual Heal Care 2018;30:161–8. 10.1093/intqhc/mzy005 29394354

[37] Faden RR, Kass NE, Goodman SN, et al An ethics framework for a learning health care system: a departure from traditional research ethics and clinical ethics. Hastings Cent Rep 2013;Spec No:S16–27. 10.1002/hast.134 23315888

[38] WHO WHO guidelines on ethical issues in public health surveillance, 2017 Available: https://www.who.int/ethics/publications/public-health-surveillance/en/ [Accessed 11 Sep 2020].

[39] Luyckx VA, Biller-Andorno N, Saxena A, et al Health policy and systems research: towards a better understanding and review of ethical issues. BMJ Glob Health 2017;2:e000314. 10.1136/bmjgh-2017-000314 PMC5717951 29225934

[40] Hyder AA, Krubiner CB, Bloom G, et al Exploring the ethics of long-term research engagement with communities in low- and middle-income countries. Public Health Ethics 2012;5:252–62. 10.1093/phe/phs012

[41] Anane-Sarpong E, Wangmo T, Sankoh O, et al Application of ethical principles to research using public health data in the global South: perspectives from Africa. Dev World Bioeth 2018;18:98–108. 10.1111/dewb.12138 28008708

[42] Aborigo RA, Allotey P, Tindana P, et al Cultural imperatives and the ethics of verbal autopsies in rural Ghana. Glob Health Action 2013;6:18570–11. 10.3402/gha.v6i0.18570 24054087 PMC3779354

[43] Bird J, Byass P, Kahn K A matter of life and death: practical and ethical constraints in the development of a mobile verbal autopsy tool BT. Proceedings of the SIGCHI Conference on Human Factors in Computing Systems, 2013:1489–98.

[44] Twine R, Hundt GL, Kahn K The ‘experimental public’ in longitudinal health research: views of local leaders and service providers in rural South Africa. Glob Health Res Policy 2017;2:26 10.1186/s41256-017-0046-7 29202094 PMC5683227

[45] Mondain N Exploring respondents’ understanding and perceptions of demographic surveillance systems in Western Africa: methodological and ethical issues. African Popul Stud 2013;24 10.11564/24-3-297

[46] Twine R, Kahn K, Journal GH-GI Assessing the effectiveness of a longitudinal knowledge dissemination intervention. epress.lib.uts.edu.au 2017.

[47] Mondain N, Delaunay V, Ouédraogo V Reporting results back in health and demographic surveillance systems (HDSS): an ethical requirement and a strategy for improving health behaviours. African Popul Stud 2016;30 10.11564/30-2-840

[48] Dunn M, Sheehan M, Hope T, et al Toward methodological innovation in empirical ethics research. Camb Q Healthc Ethics 2012;21:466–80. 10.1017/S0963180112000242 22828041

[49] Kon AA The role of empirical research in bioethics. Am J Bioeth 2009;9:59–65. 10.1080/15265160902874320 PMC2826359 19998120

[50] INDEPTH Network MEMBER HDSSs, 2020 Available: http://www.indepth-network.org/member-centres [Accessed 10 Sep 2020].

[51] Hammersley M, Atkinson P Ethnography: principles in practice. Third Edition London: Routledge, 2007.

[52] Liu F, Maitlis S Nonparticipant Observation : Mills AJ, Durepos G, Wiebe E, Encyclopedia of case study research. Thousand Oaks, CA: SAGE Publ, 2010: 610–2. https://books.google.co.ke/books?hl=en&lr=&id=EMBDWriZDQ8C&oi=fnd&pg=PP1&dq=Liu,+F.,+%26+Maitlis,+S.+(2010).+Nonparticipant+Observation.+In+Albert+J.+Mills,+G.+Durepos,+%26+E.+Wiebe+(Eds.),+Encyclopedia+of+Case+Study+Research.+(pp.+610-612).+Thousand+Oaks

[53] Gutmann J Qualitative research practice: a guide for social science students and researchers (2nd EDN). Int. J. Market Res. 2014;56:407 10.2501/IJMR-2014

[54] Herbst K, Juvekar S, Bhattacharjee T, et al The indepth data Repository. J Empir Res Hum Res Ethics 2015;10:324–33. 10.1177/1556264615594600 26297754 PMC4547208

[55] Baiden F, Bawah A, Biai S, et al Setting international standards for verbal autopsy. Bull World Health Organ 2007;85:570–1. 10.2471/BLT.07.043745 17768508 PMC2636382

[56] Leitao J, Chandramohan D, Byass P, et al Revising the WHO verbal autopsy instrument to facilitate routine cause-of-death monitoring. Glob Health Action 2013;6:21518. 10.3402/gha.v6i0.21518 24041439 PMC3774013

[57] Klingler C, Barrett DH, Ondrusek N, et al Beyond research ethics: novel approaches of 3 major public health institutions to provide ethics input on public health practice activities. J Public Health Manag Pract 2020;26:E12-E22. 10.1097/PHH.0000000000000734 29481545 PMC6650371

[58] Baum NM, Gollust SE, Goold SD, et al Looking ahead: addressing ethical challenges in public health practice. J Law Med Ethics 2007;35:657–67. 10.1111/j.1748-720X.2007.00188.x 18076516

[59] Salerno J, Peters ES, Pinney SM, et al Untangling the ethical intersection of epidemiology, human subjects research, and public health. Ann Epidemiol 2019;34:1–5. 10.1016/j.annepidem.2019.03.009 31005553 PMC7370238

[60] Tindana PO, Singh JA, Tracy CS, et al Grand challenges in global health: community engagement in research in developing countries. PLoS Med 2007;4:e273–5. 10.1371/journal.pmed.0040273 17850178 PMC1989740

[61] Participants in the Community Engagement and Consent Workshop, Kilifi, Kenya, March 2011 Consent and community engagement in diverse research contexts. J Empir Res Hum Res Ethics 2013;8:1–18. 10.1525/jer.2013.8.4.1 PMC4836561 24169417

[62] Montgomery CM, Pool R From ‘trial community’ to ‘experimental publics’: how clinical research shapes public participation. Crit Public Health 2017;27:50–62. 10.1080/09581596.2016.1212161

[63] Kingori P Experiencing everyday ethics in context: frontline data collectors perspectives and practices of bioethics. Soc Sci Med 2013;98:361–70. 10.1016/j.socscimed.2013.10.013 24210881 PMC3898703

[64] Molyneux CS, Peshu N, Marsh K Trust and informed consent: insights from community members on the Kenyan coast. Soc Sci Med 2005;61:1463–73. 10.1016/j.socscimed.2004.11.073 16005781

[65] Baiden F, Akazili J, Chatio S, et al Should consent forms used in clinical trials be translated into the local dialects? A survey among past participants in rural Ghana. Clin Trials 2016;13:234–9. 10.1177/1740774515609290 26452387

[66] Lee LM, Heilig CM, White A Ethical justification for conducting public health surveillance without patient consent. Am J Public Health 2012;102:38–44. 10.2105/AJPH.2011.300297 22095338 PMC3490562

[67] Crampin AC, Dube A, Mboma S, et al Profile: the Karonga health and demographic surveillance system. Int J Epidemiol 2012;41:676–85. 10.1093/ije/dys088 22729235 PMC3396313

[68] Derra K, Rouamba E, Kazienga A, et al Profile: Nanoro health and demographic surveillance system. Int J Epidemiol 2012;41:1293–301. 10.1093/ije/dys159 23045201

[69] Sacoor C, Nhacolo A, Nhalungo D, et al Profile: Manhiça health research centre (Manhiça HDSS). Int J Epidemiol 2013;42:1309–18. 10.1093/ije/dyt148 24159076

[70] Wanyua S, Ndemwa M, Goto K, et al Profile: the Mbita health and demographic surveillance system. Int J Epidemiol 2013;42:1678–85. 10.1093/ije/dyt180 24415606

[71] Bull S, Roberts N, Parker M Views of ethical best practices in sharing individual-level data from medical and public health research: a systematic scoping review. J Empir Res Hum Res Ethics 2015;10:225–38. 10.1177/1556264615594767 26297745 PMC4548478

[72] van Panhuis WG, Paul P, Emerson C, et al A systematic review of barriers to data sharing in public health. BMC Public Health 2014;14:1144. 10.1186/1471-2458-14-1144 25377061 PMC4239377

[73] Chawinga WD, Zinn S Global perspectives of research data sharing: a systematic literature review. Libr Inf Sci Res 2019;41:109–22. 10.1016/j.lisr.2019.04.004

[74] Bezuidenhout LM, Leonelli S, Kelly AH, et al Beyond the digital divide: towards a situated approach to open data. Sci Public Policy 2017;44:464–75. 10.1093/scipol/scw036

[75] Ghafur T, Islam MM, Alam N, et al Health and demographic surveillance system sites: reflections on global health research ethics. J Popul Soc Stud 2020;28:265–75. 10.25133/JPSSv28n3.018

[76] United Nations Impact of COVID-19 — UN legal identity agenda, 2020 Available: https://unstats.un.org/legal-identity-agenda/COVID-19/ [Accessed 11 Sep 2020].

[77] Nuffield Council on Bioethics Research in global health emergencies: ethical issues; 2020.

[78] INDEPTH Network,, African Institute for Development Policy The Contribution of the INDEPTH Network to Malaria :A Synthesis of Research Evidence Published from 1998 to 2009 - Research Inbrief; 2012.

[79] Streatfield PK, Khan WA, Bhuiya A, et al HIV/AIDS-related mortality in Africa and Asia: evidence from indepth health and demographic surveillance system sites. Glob Health Action 2014;7:25370. 10.3402/gha.v7.25370 25377330 PMC4220131

[80] Arthur SS, Nyide B, Soura AB, et al Tackling malnutrition: a systematic review of 15-year research evidence from indepth health and demographic surveillance systems. Glob Health Action 2015;8:28298. 10.3402/gha.v8.28298 26519130 PMC4627942

[81] Gerritsen A, Bocquier P, White M, et al Health and demographic surveillance systems: contributing to an understanding of the dynamics in migration and health. Glob Health Action 2013;6:21496. 10.3402/gha.v6i0.21496 23849188 PMC3710398

[82] Hondula DM, Rocklöv J, Sankoh OA Past, present, and future climate at select indepth member health and demographic surveillance systems in Africa and Asia. Glob Health Action 2012;5:19083. 10.3402/gha.v5i0.19083 PMC3508753 23195511

[83] Coates MM, Kamanda M, Kintu A, et al A comparison of all-cause and cause-specific mortality by household socioeconomic status across seven indepth network health and demographic surveillance systems in sub-Saharan Africa. Glob Health Action 2019;12:1608013. 10.1080/16549716.2019.1608013 31092155 PMC6534200

[84] Pisani E, Kok M In the eye of the beholder: to make global health estimates useful, make them more socially robust. Glob Health Action 2017;10:1266180. 10.3402/gha.v9.32298 28532303 PMC5124117

[85] Hanney SR, Gonzalez-Block MA, Buxton MJ, et al The utilisation of health research in policy-making: concepts, examples and methods of assessment. Health Res Policy Syst 2003;1:2. 10.1186/1478-4505-1-2 12646071 PMC151555

[86] Piasecki J, Waligora M, Dranseika V What do ethical guidelines for epidemiology say about an ethics review? A qualitative systematic review. Sci Eng Ethics 2017;23:743–68. 10.1007/s11948-016-9829-3 27848192 PMC5486592

[87] Tsoka-Gwegweni JM, Wassenaar DR Using the Emanuel et al. framework to assess ethical issues raised by a biomedical research ethics committee in South Africa. J Empir Res Hum Res Ethics 2014;9:36–45. 10.1177/1556264614553172 25747689 PMC8285026

[88] Azétsop J, Rennie S Principlism, medical individualism, and health promotion in resource-poor countries: can autonomy-based bioethics promote social justice and population health? Philos Ethics Humanit Med 2010;5:1. 10.1186/1747-5341-5-1 20082703 PMC2828974

[89] Ferreira Antunes JL A dictionary of epidemiology. J Epidemiol Community Health 2009;63:337. 10.1136/jech.2008.082511 19366887

[90] Pearce N Classification of epidemiological study designs. Int J Epidemiol 2012;41:393–7. 10.1093/ije/dys049 22493323

[91] Krishnan A What are academic disciplines? some observations on the Disciplinarity vs. interdisciplinarity debate 2009.

[92] Rubel A Justifying public health surveillance: basic interests, unreasonable exercise, and privacy. Kennedy Inst Ethics J 2012;22:1–33. 10.1353/ken.2012.0001 22787956

[93] Ondrusek NK, Willison DJ, Haroun V, et al A risk screening tool for ethical appraisal of evidence-generating initiatives. BMC Med Ethics 2015;16:47. 10.1186/s12910-015-0039-3 26149410 PMC4494793

[94] Doshi P WHO's malaria vaccine study represents a "serious breach of international ethical standards". BMJ 2020;368:m734. 10.1136/bmj.m734 32102785

[95] Weijer C The WHO malaria vaccine trial: a bioethicist responds. The BMJ 2020 https://www.bmj.com/content/368/bmj.m734/rr-5

[96] Swaminathan S, O’Brien K, Alonso P The WHO malaria vaccine implementation program: Clarifying misconceptions. The BMJ 2020 https://www.bmj.com/content/368/bmj.m734/rr-1

[97] Smith B Generalizability in qualitative research: misunderstandings, opportunities and recommendations for the sport and exercise sciences. Qual Res Sport Exerc Health 2018;10:137–49. 10.1080/2159676X.2017.1393221

[98] Owusu-Agyei S, Nettey OEA, Zandoh C, et al Demographic patterns and trends in central Ghana: baseline indicators from the Kintampo health and demographic surveillance system. Glob Health Action 2012;5:19033. 10.3402/gha.v5i0.19033 PMC3529298 23273249

[99] Oduro AR, Wak G, Azongo D, et al Profile of the Navrongo health and demographic surveillance system. Int J Epidemiol 2012;41:968–76. 10.1093/ije/dys111 22933645

[100] Beguy D, Elung'ata P, Mberu B, et al Health & Demographic Surveillance System Profile: The Nairobi Urban Health and Demographic Surveillance System (NUHDSS). Int J Epidemiol 2015;44:462–71. 10.1093/ije/dyu251 25596586

