# Supplementary material for: Towards an appropriate ethics framework for Health and Demographic Surveillance Systems (HDSS): learning from issues faced in diverse HDSS in sub-Saharan Africa
Source: BMJ Glob Health. 2021 Jan 6;6(1):e004008. doi: 10.1136/bmjgh-2020-004008 (PMC7789450; doi:10.1136/bmjgh-2020-004008)
Supplement: Supplementary data [file bmjgh-2020-004008supp001.pdf]

Rationale for establishment of health and demographic surveillance systems across sub-Saharan Africa

Country	HDSS Site	Site-specific Rationale for establishment
**Kenya**	Kilifi	To create a longitudinal community-based study linked to hospital morbidity surveillance in order to define the incidence and prevalence of local diseases of childhood, evaluate the impact of community-based interventions against infectious diseases and to provide an epidemiological sampling frame for studies KEMRI Wellcome Trust[1].
	Nairobi	To provide a platform to investigate the long-term social, economic and health consequences of urban residence, and to serve as a primary research tool for intervention and impact evaluation studies focusing on the needs of the urban poor in sub-Saharan Africa[2,3].
	Kisumu	To support a large insecticide-treated bed net trial. HDSS sustained post-trial to provide multi-disciplinary research data[4].
	Kombewa	To support regulated clinical trials, nested studies and local disease surveillance, by integrating household and health-facility-based data[5].
	Mbita	To establish baseline data on health, demographic, socio-economic and environmental characteristics of communities in Mbita District. To evaluate impact of health and socioeconomic interventions, support research on communicable and non-communicable diseases, and provide platform for education and training[6].
**Ghana**	Dodowa	Established to enable registration of households into a pilot community health insurance scheme (2003). Could not be sustained after initial census due to lack of funding. With research funding in 2005, re-established to provide platform for large scale trials and surveillance[7].
	Kintampo	To research diseases of public health importance nationally and internationally and contribute to reducing these diseases by developing interventions and sharing data with ministry of health and other health organisations.
	Navrongo	Established to support evaluation of a permethrin impregnated bed net trial. Preceded by surveillance activities that focussed on children <5years as part of the Ghana Vitamin A Supplementation Trial[8].
**Uganda**	Iganga/Mayuge	Established as a research setting to provide data for informing local and global policy.<br>To serve as platform for developing operational research capacity for graduate students and academic staff at Makerere University, and to generate continuous population data for evidence-based policymaking at district and national level[9].
	Kyamulibwa	To examine trends in prevalence and incidence of HIV infection and their determinants[10].
	Rakai	To support population studies on HIV/AIDS[11].
**Malawi**	Karonga	To provide a platform for epidemiological studies of HIV and HIV-associated infectious disease and to monitor the impact of interventions[12].
**Burkina Faso**	Kaya	To regularly generate quality data for assessing population health, evaluating health interventions and supporting research[13].
	Nanoro	To provide a highly standardized means of monitoring the population living within the catchment area of a district hospital in Nanoro and to

		support a large pharmacovigilance study on artemisinin-based combination treatments (ACT) and a phase 3 vaccine trial[14].
	Nouna	To provide reliable demographic and health indicators for a rural population in Burkina Faso, and to generate an infrastructure and a sampling frame for epidemiological and clinical studies nested into this dynamic cohort[15].
	Oagadougou	To address the problems of the urban poor, by collecting data on the lives of people in one informal and one formal neighbourhood in Oagadougou[16].
**Cote d'Ivoire**	Taabo	To serve as a platform for evaluating interventions and health system strengthening with the aim of reducing morbidity and mortality especially from malaria and NTDs in Taabo, Cote d'Ivoire and sub-Saharan Africa. To conduct interdisciplinary research and build capacity in health research[17].
**Ethiopia**	Arba Minch	To track demographic changes, including births, deaths, migrations and marriages[18].
	Butajira	To develop and evaluate a system for continuous registration of births and deaths, to generate valid data on fertility and mortality and to provide a population and study base for essential health research and intervention in the area.
	Dabat	To generate longitudinal data on health and population at district level and provide a study base and sampling frame for community-based research.
	Kersa	To create a framework for research at the community level, given the lack of reliable information about health problems of public health importance[19].
	Kilite Awlaelo	To generate longitudinal health and demographic data for a rural low-income population[20].
**Gambia**	Farafenni	To generate health and demographic data for the evaluation of a primary health care programme by The Gambian government[21].
	West Kiang	To support research and provision of healthcare services[22].
**Guinea Bissau**	Bandim	To assess the prevalence of neonatal tetanus and to collect data on child mortality to monitor the impact of a Danish International Development Agency (DANIDA)-sponsored project to strengthen primary healthcare[23].
	Manhica	To provide data on fertility, migration and mortality and support epidemiological studies of infectious diseases for children < 5yrs and pregnant women[24].
**Nigeria**	Nahuche	To support research for informing policy health service delivery[25].
	Cross River	To generate timely and reliable data to inform policy makers on differences in population characteristics and access to health services[26].
**Senegal**	Bandafassi	To study the health and demographic situation of a rural West African population and assess changes over time, including causes of these changes. Preceded by a genetic study that involved collection of blood samples and demographic information to investigate survival rates in various genotype sub-groups in the population[27].
	Mlomp	To provide health and demographic data for a rural population that differed from that in Niakhar and Bandafassi in terms of historic, economic and ethnic characteristics, and therefore enhance the

		coverage of the diverse demographic and epidemiological characteristics in Senegal[28].
	Niakhar	To demonstrate the capacity to record reliable demographic and epidemiological data in rural areas of Africa. Because previous population-based data collection and civil registration had excluded African populations in rural areas[29].
**South Africa**	Agincourt	To provide reliable population-based information for programme planning and resource allocation towards strengthening the district health system[30].
	AHRI	To provide high quality longitudinal data for monitoring population health and demographic changes as a result of the HIV epidemic and to evaluate interventions against HIV[31].
	DIMAMO (formerly Dikgale)	To serve as a sampling framework for monitoring non-communicable diseases and their risk factors, given concerns in the 1990's that exposures to risk factors for NCDs in South Africa were increasing with changing lifestyles[32].
**Tanzania**	Ifakara	To support a field trial on effectiveness of social marketing of bed nets on morbidity and mortality of children aged under 5 years[33].
	Rufiji	To evaluate the impact, on morbidity and mortality, of a policy that introduced evidence-based health planning. Evaluate impact of changes in health polices and services[34].
	Magu	To monitor mortality, fertility and mobility in the general population as part of an open cohort HIV study[35].

References

1 Scott JAG, Bauni E, Moisi JC, *et al.* Profile: The Kilifi Health and Demographic Surveillance System (KHDSS). *Int J Epidemiol* 2012;**41**:650–7. doi:10.1093/ije/dys062

2 Beguy D, Elung'ata P, Mberu B, *et al.* Health & Demographic Surveillance System Profile: The Nairobi Urban Health and Demographic Surveillance System (NUHDSS). *Int J Epidemiol* 2015;**44**:462–71. doi:10.1093/ije/dyu251

3 Emina J, Beguy D, Zulu EM, *et al.* Monitoring of Health and Demographic Outcomes in Poor Urban Settlements: Evidence from the Nairobi Urban Health and Demographic Surveillance System. *J Urban Heal* 2011;**88**:200–18. doi:10.1007/s11524-011-9594-1

4 Odhiambo FO, Laserson KF, Sewe M, *et al.* Profile: The KEMRI/CDC Health and Demographic Surveillance System--Western Kenya. *Int J Epidemiol* 2012;**41**:977–87. doi:10.1093/ije/dys108

5 Sifuna P, Oyugi M, Ogutu B, *et al.* Health & Demographic Surveillance System Profile: The Kombewa Health and Demographic Surveillance System (Kombewa HDSS). *Int J Epidemiol* 2014;**43**:1097–104. doi:10.1093/ije/dyu139

6 Wanyua S, Ndemwa M, Goto K, *et al.* Profile: The Mbita Health and Demographic Surveillance System. *Int J Epidemiol* 2013;**42**:1678–85. doi:10.1093/ije/dyt180

7 Gyapong M, Sarpong D, Awini E, *et al.* Profile: The Dodowa HDSS. *Int J Epidemiol* 2013;**42**:1686–96. doi:10.1093/ije/dyt197

8 Oduro AR, Wak G, Azongo D, *et al.* Profile of the Navrongo health and demographic surveillance system. *Int J Epidemiol* 2012;**41**:968–76. doi:10.1093/ije/dys111

9 Kadobera D, Waiswa P, Peterson S, *et al.* Comparing performance of methods used to identify

pregnant women, pregnancy outcomes, and child mortality in the Iganga-Mayuge Health and Demographic Surveillance Site, Uganda. *Glob Health Action* 2017;**10**:1356641. doi:10.1080/16549716.2017.1356641

10 Asiki G, Murphy G, Nakiyingi-Miiro J, *et al.* Data resource profile: The general population cohort in rural southwestern Uganda: A platform for communicable and non-communicable disease studies. *Int J Epidemiol* 2013;**42**:129–41. doi:10.1093/ije/dys234

11 Wawer MJ, Gray RH, Sewankambo NK, *et al.* A randomized, community trial of intensive sexually transmitted disease control for AIDS prevention, Rakai, Uganda. *AIDS* 1998;**12**:1211–25. doi:10.1097/00002030-199810000-00014

12 Crampin AC, Dube A, Mboma S, *et al.* Profile: The Karonga health and demographic surveillance system. *Int J Epidemiol* 2012;**41**:676–85. doi:10.1093/ije/dys088

13 Kouanda S, Bado A, Yaméogo M, *et al.* The Kaya HDSS, Burkina Faso: a platform for epidemiological studies and health programme evaluation. *Int J Epidemiol* 2013;**42**:741–9. doi:10.1093/ije/dyt076

14 Derra K, Rouamba E, Kazienga A, *et al.* Profile: Nanoro health and demographic surveillance system. *Int J Epidemiol* 2012;**41**:1293–301. doi:10.1093/ije/dys159

15 Sié A, Louis V, Gbangou A, *et al.* The Health and Demographic Surveillance System (HDSS) in Nouna, Burkina Faso, 1993–2007. *Glob Health Action* 2010;**3**:5284. doi:10.3402/gha.v3i0.5284

16 Rossier C, Soura A, Baya B, *et al.* Profile: The Ouagadougou Health and Demographic Surveillance System. *Int J Epidemiol* 2012;**41**:658–66. doi:10.1093/ije/dys090

17 Koné S, Baikoro N, N'Guessan Y, *et al.* Health & Demographic Surveillance System Profile: The Taabo Health and Demographic Surveillance System, Côte d'Ivoire. *Int J Epidemiol* 2015;**44**:87–97. doi:10.1093/ije/dyu221

18 Gultie T, Ayele G, Tariku B, *et al.* Trend of declining bed net utilization among pregnant women in Ethiopia: new data from the Arba Minch Health and Demographic Surveillance System, 2010–2016. *Malar J* 2020;**19**:142. doi:10.1186/s12936-020-03211-x

19 Assefa N, Oljira L, Baraki N, *et al.* HDSS Profile: The Kersa Health and Demographic Surveillance System. *Int J Epidemiol* 2016;**45**:94–101. doi:10.1093/ije/dyv284

20 Weldearegawi B, Spigt M, Berhane Y, *et al.* Mortality Level and Predictors in a Rural Ethiopian Population: Community Based Longitudinal Study. *PLoS One* 2014;**9**:e93099. doi:10.1371/journal.pone.0093099

21 Jasseh M, Gomez P, Greenwood BM, *et al.* Health & Demographic Surveillance System Profile: Farafenni Health and Demographic Surveillance System in The Gambia. *Int J Epidemiol* 2015;**44**:837–47. doi:10.1093/ije/dyv049

22 Hennig BJ, Unger SA, Dondeh BL, *et al.* Cohort Profile: The Kiang West Longitudinal Population Study (KWLPS)—a platform for integrated research and health care provision in rural Gambia. *Int J Epidemiol* 2015;**46**:dyv206. doi:10.1093/ije/dyv206

23 Thysen SM, Fernandes M, Benn CS, *et al.* Cohort profile : Bandim Health Project's (BHP) rural Health and Demographic Surveillance System (HDSS)—a nationally representative HDSS in Guinea-Bissau. *BMJ Open* 2019;**9**:e028775. doi:10.1136/bmjopen-2018-028775

24 Sacoor C, Nhacolo A, Nhalungo D, *et al.* Profile: Manhica Health Research Centre (Manhica HDSS). *Int J Epidemiol* 2013;**42**:1309–18. doi:10.1093/ije/dyt148

25 Alabi O, Doctor H V., Jumare A, *et al.* Health & Demographic Surveillance System Profile: The Nahuche Health and Demographic Surveillance System, Northern Nigeria (Nahuche HDSS). *Int J Epidemiol* 2014;**43**:1770–80. doi:10.1093/ije/dyu197

26 Arikpo II, Okoro A, Esu E, *et al.* Differences in Population Dynamics and Uptake of Reproductive Health Services in the Urban and Rural Cohorts of Cross River Health and Demographic Surveillance System of Southern Nigeria. *Dev Ctries Stud* 2019;**9**. doi:10.7176/DCS

27 Pison G, Douillot L, Kante AM, *et al.* Health & demographic surveillance system profile: Bandafassi health and demographic surveillance system (Bandafassi HDSS), Senegal. *Int J Epidemiol* 2014;**43**:739–48. doi:10.1093/ije/dyu086

28 Pison G, Beck B, Ndiaye O, *et al.* HDSS Profile: Mlomp Health and Demographic Surveillance System (Mlomp HDSS), Senegal. *Int J Epidemiol* 2018;**47**:1025–33. doi:10.1093/ije/dyy075

29 Delaunay V, Douillot L, Diallo A, *et al.* Profile: The niakhar health and demographic surveillance system. *Int J Epidemiol* 2013;**42**:1002–11. doi:10.1093/ije/dyt100

30 Kahn K, Collinson MA, Xavier G??mez-oliv?? F, *et al.* Profile: Agincourt health and socio-demographic surveillance system. *Int J Epidemiol* 2012;**41**:988–1001. doi:10.1093/ije/dys115

31 Tanser F, Hosegood V, Barnighausen T, *et al.* Cohort Profile: Africa Centre Demographic Information System (ACDIS) and population-based HIV survey. *Int J Epidemiol* 2008;**37**:956–62. doi:10.1093/ije/dym211

32 Alberts M, Dikotope SA, Choma SR, *et al.* Health & Demographic Surveillance System Profile: The Dikgale Health and Demographic Surveillance System. *Int J Epidemiol* 2015;**44**:1565–71. doi:10.1093/ije/dyv157

33 Geubbels E, Amri S, Levira F, *et al.* Health & Demographic Surveillance System Profile: The Ifakara Rural and Urban Health and Demographic Surveillance System (Ifakara HDSS). *Int J Epidemiol* 2015;**44**:848–61. doi:10.1093/ije/dyv068

34 Mrema S, Kante AM, Levira F, *et al.* Health & Demographic Surveillance System Profile: The Rufiji Health and Demographic Surveillance System (Rufiji HDSS). *Int J Epidemiol* 2015;**44**:472–83. doi:10.1093/ije/dyv018

35 Kishamawe C, Isingo R, Mtenga B, *et al.* Health & Demographic Surveillance System Profile: The Magu Health and Demographic Surveillance System (Magu HDSS). *Int J Epidemiol* 2015;**44**:1851–61. doi:10.1093/ije/dyv188
